# Nuclear-Targeted Deleted in Liver Cancer 1 (DLC1) Is Less Efficient in Exerting Its Tumor Suppressive Activity Both *In Vitro* and *In Vivo*

**DOI:** 10.1371/journal.pone.0025547

**Published:** 2011-09-26

**Authors:** Lo-Kong Chan, Frankie Chi Fat Ko, Karen Man-Fong Sze, Irene Oi-Lin Ng, Judy Wai Ping Yam

**Affiliations:** 1 Department of Pathology, Li Ka Shing Faculty of Medicine, The University of Hong Kong, Hong Kong; 2 State Key Laboratory for Liver Research, The University of Hong Kong, Hong Kong; 3 Centre for Cancer Research, Li Ka Shing Faculty of Medicine, The University of Hong Kong, Hong Kong; Baylor College of Medicine, United States of America

## Abstract

**Background:**

Deleted in liver cancer 1 (DLC1) serves as an important RhoGTPase activating protein (RhoGAP) protein that terminates active RhoA signaling in human cancers. Increasing evidence has demonstrated that the tumor suppressive activity of DLC1 depends not only on RhoGAP activity, but also relies on proper focal adhesion localization through its interaction with tensin family proteins. Recently, there are reports showing that DLC1 can also be found in the nucleus; however, the existence and the relative tumor suppressive activity of nuclear DLC1 have never been clearly addressed.

**Methodology and Principal Findings:**

We herein provide new evidence that DLC1 protein, which predominantly associated with focal adhesions and localized in cytosol, dynamically shuttled between cytoplasm and nucleus. Treatment of cells with nuclear export blocker, Leptomycin B (LMB), retained DLC1 in the nucleus. To understand the nuclear entry of DLC1, we identified amino acids 600–700 of DLC1 as a novel region that is important for its nuclear localization. The tumor suppressive activity of nuclear DLC1 was directly assessed by employing a nuclear localization signal (NLS) fusion variant of DLC1 (NLS-DLC1) with preferential nuclear localization. In SMMC-7721 HCC cells, expression of NLS-DLC1 failed to suppress colony formation and actin stress fiber formation *in vitro*. The abrogated tumor suppressive activity of nuclear DLC1 was demonstrated for the first time *in vivo* by subcutaneously injecting p53^−/−^ RasV12 hepatoblasts with stable NLS-DLC1 expression in nude mice. The injected hepatoblasts with NLS-DLC1 expression effectively formed tumors when compared with the non-nuclear targeted DLC1.

**Conclusions/Significance:**

Our study identified a novel region responsible for the nuclear entry of DLC1 and demonstrated the functional difference of DLC1 in different cellular compartments both *in vitro* and *in vivo*.

## Introduction

Deleted in Liver Cancer 1 (DLC1) was first cloned by subtractive hybridization as a gene fragment that was frequently deleted in human hepatocellular carcinoma (HCC) [1]. Accumulating evidence in the last decade has supported that DLC1 is underexpressed in various kinds of human cancers besides HCC [2], [3], [4], [5], [6], [7]. Restoration of DLC1 by ectopic expression in cells with low or no endogenous expression of DLC1 retards cell proliferation, induces apoptosis, slows down cell movement and disintegrates actin cytoskeletal structure *in vitro* [8], [9], [10]. Conversely, DLC1 knockdown promotes tumorigenesis of a Myc driven *in vivo* mouse hepatocarcinogenesis model with a p53 null background [11]. The nature of the frequent pathological underexpression of DLC1 and the robust experimental tumor suppressive activity both strongly support DLC1 functions as a *bona fide* tumor suppressor.

The tumor suppressive activity of DLC1 is tightly linked to its intrinsic RhoGAP activity, which down-regulates the Rho-mediated biological response. DLC1 has been found to be RhoA-, B-, C- and CDC42-specific [12], [13]. Focal adhesion localization through interaction with tensin family protein is one of the characteristics of DLC1 and is functionally associated with its tumor suppressive activity. This was supported by the evidence that DLC1 mutant with intact RhoGAP domain but failed to be targeted to the focal adhesions exhibited reduced growth suppressive activity *in vitro* [10], [14]. Since the focal adhesion targeting sites in DLC1 overlap with the tensin protein binding site, it has been accepted that DLC1 couples with tensin and functions as tumor suppressive complex in terminating the focal adhesion-associated Rho activity [10], [14], [15].

A recent study has suggested the presence of basic residues rich motif in DLC1 resembling the NLS [16]. Also, it has been suggested that the serine/threonine specific protein kinase D phosphorylates DLC1 to create a 14-3-3 docking site. Complex formation with 14-3-3 sequesters DLC1 in the cytoplasm and stops its nuclear entry [17]. However, the existence and regulation of the nuclear DLC1 have not been thoroughly explored. Most importantly, the relative tumor suppressive activity of nuclear DLC1 has never been directly addressed. In this study, we have provided comprehensive evidence that DLC1 protein constantly shuttles between the cytoplasm and nucleus. We have performed the first functional characterization of nuclear targeted DLC1 to examine its basic and tumor suppressive activity both *in vitro* and *in vivo*. By studying the subcellular localization of various DLC1 mutants, we have identified a novel region within the RhoGAP domain which is important and sufficient for this nuclear import.

## Results

### DLC1 shuttled between cytoplasm and nucleus

**Figure 1:**
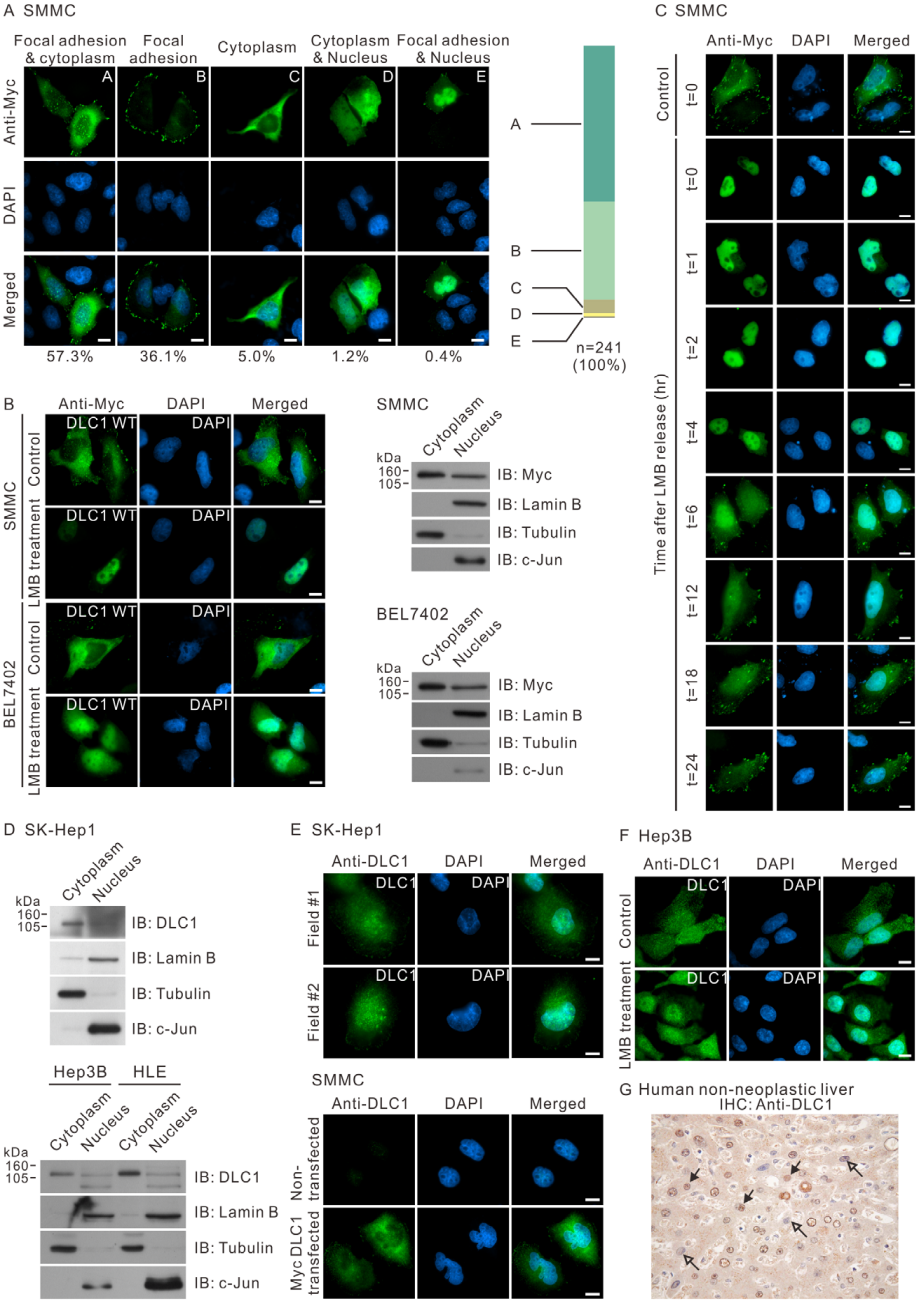
DLC1 shuttled between cytoplasm and nucleus. (**A**) SMMC-7721 cells were transiently transfected with Myc-tagged DLC1. DLC1 was visualized with anti-Myc antibody followed by FITC conjugated secondary antibody. Nucleus was counterstained with DAPI. Subcellular localization pattern of DLC1 of 241 DLC1 transfected cells was counted and categorized into group **A** to **E** as indicated. (**B**) SMMC-7721 and BEL7402 cells were transiently transfected with Myc-tagged DLC1 and subjected to LMB treatment followed by immunofluorescence staining with anti-Myc antibody (*left panel*). The transfected cells were fractionated into cytoplasmic and nuclear portions followed by immunoblotting against Myc for exogenous DLC1, tubulin (cytoplasmic marker), LaminB and c-jun (nuclear marker) (*right panel*). (**C**) SMMC-7721 cells were transiently transfected with Myc-tagged DLC1, followed by LMB treatment for 6 hours. After LMB treatment (which was designated as t = 0), cells were cultured with fresh medium and were fixed at the indicated time point. Exogenous DLC1 was visualized by anti-Myc antibody. (**D**) Hep3B, HLE cells and SK-Hep1 cells were fractionated into cytoplasmic and nuclear portions followed by immunoblotting using antibodies against endogenous DLC1, tubulin, LaminB and c-jun. (**E**) Endogenous DLC1 in SK-Hep1 cells was visualized by polyclonal anti-DLC1 antibody, followed by FITC-conjugated secondary antibody. To serve as a negative control, SMMC-7721 cells without DLC1 expression was stained with the polycloncal anti-DLC1 antibody. The DLC1 antibody could only detect signal in SMMC-7721 cells with exogenous DLC1 overexpression but not the non-transfected cells. (**F**) Hep3B cells were treated with LMB for 6 hours. Endogenous DLC1 was then visualized by polyclonal anti-DLC1 antibody, followed by FITC-conjugated secondary antibody. (**G**) A section of human non-neoplastic liver was immunostained for DLC1 (Brown). The nuclei were counterstained by hematoxylin (Blue). Cells showing positive nuclear DLC1 staining were indicated by the solid-headed arrows. Cell nucleus showing negative DLC1 staining was indicated by the empty-headed arrows. Scale bar: 10 µm.

Expression of Myc-tagged DLC1 (Myc-DLC1) in SMMC-7721 cells revealed its existence in focal adhesions, cytoplasm and nucleus (**Fig. 1A**). Quantitative examination of DLC1 expression in cells showed its dominant cytoplasmic localization with punctate focal adhesion pattern at the cell periphery. To explore the movement of DLC1 between the cytoplasm and nucleus, Myc-DLC1 localization in cells was studied in cells treated with exportin blocker, LMB. Treatment with LMB resulted in the nuclear retention of Myc-DLC1 in SMMC-7721 and BEL7402 cells (**Fig. 1B** **,** ***left panel***). Notably, focal adhesion localized DLC1 was still observed, implicating only the non-focal adhesion localized DLC1 was shuttled and retained in the nucleus. Cellular fractionation further corroborated the presence of DLC1 in the nuclear fraction of cell lysate (**Fig. 1B** **,** ***right panel***). Similar observation was obtained with GFP-tagged DLC1 (GFP-DLC1) (data not shown). The dynamic movement of DLC1 was observed under the withdrawal of LMB at different time points (**Fig. 1C**). Re-patterning of cytoplasmic and focal adhesion was occurred within 24 hours. We extended our investigation by examining the localization of endogenous DLC1 in various cell lines. Subcellular fractionation of Hep3B, HLE and SK-Hep1 cells followed by immunoblotting using DLC1-specific antibody supports the presence of DLC1 in the nucleus (**Fig. 1D**). Immunofluorescence for endogenous DLC1 in SK-Hep1 cells showed similar subcellular localization in the cytoplasm and nucleus (**Fig. 1E**). Consistently, endogenous DLC1 in Hep3B was retained in the nucleus after LMB treatment (**Fig. 1F**). To support the notion that DLC1 shuttles between cytoplasm and nucleus under physiological condition, we performed immunohistochemistry against DLC1 on a human non-neoplastic liver (**Fig. 1G**). Positive DLC1 staining was detected in both cytoplasm and nucleus, further supporting that DLC1 is present in the nucleus.

### DLC1 600–700 residues cooperated with NLS in the regulation of DLC1 nuclear entry

Proteins of large molecular size possess nuclear transport signals for active transport across the nuclear envelope with the aid of nuclear transport complex. It has been proposed that a monopartite NLS (423–431) [17] and bipartite NLS (415–431) [16] are present in DLC1 which facilitate the nuclear entry of DLC1. To specifically address the localization of DLC1 without the proposed NLS upon LMB treatment, we constructed DLC1Δ415–431 mutant specifically lacking the proposed NLS (**Fig. S1A and S1B**). Expression of DLC1Δ415–431 in SMMC-7721 cells showed the same subcellular localization as wildtype DLC1. Surprisingly, DLC1Δ415–431 was still sensitive to LMB treatment and was retained in the nucleus as effectively as wildtype DLC1 (**Fig. S1C**). It has also been reported that phosphorylation at serine-431 (S431) of DLC1 by protein kinase D (PKD) [17] facilitates binding between DLC1 and 14-3-3 proteins, thus preventing nuclear entry of DLC1. However, we found that DLC1 S431 phospho-defective mutant, S431A showed similar degree of nuclear retention after LMB treatment (**Fig. S1D**). Taken together, we could not provide sufficient evidence in supporting the functionality of the proposed NLS as well as the role of S431 phosphorylation in regulating the nuclear transport of DLC1.

**Figure 2:**
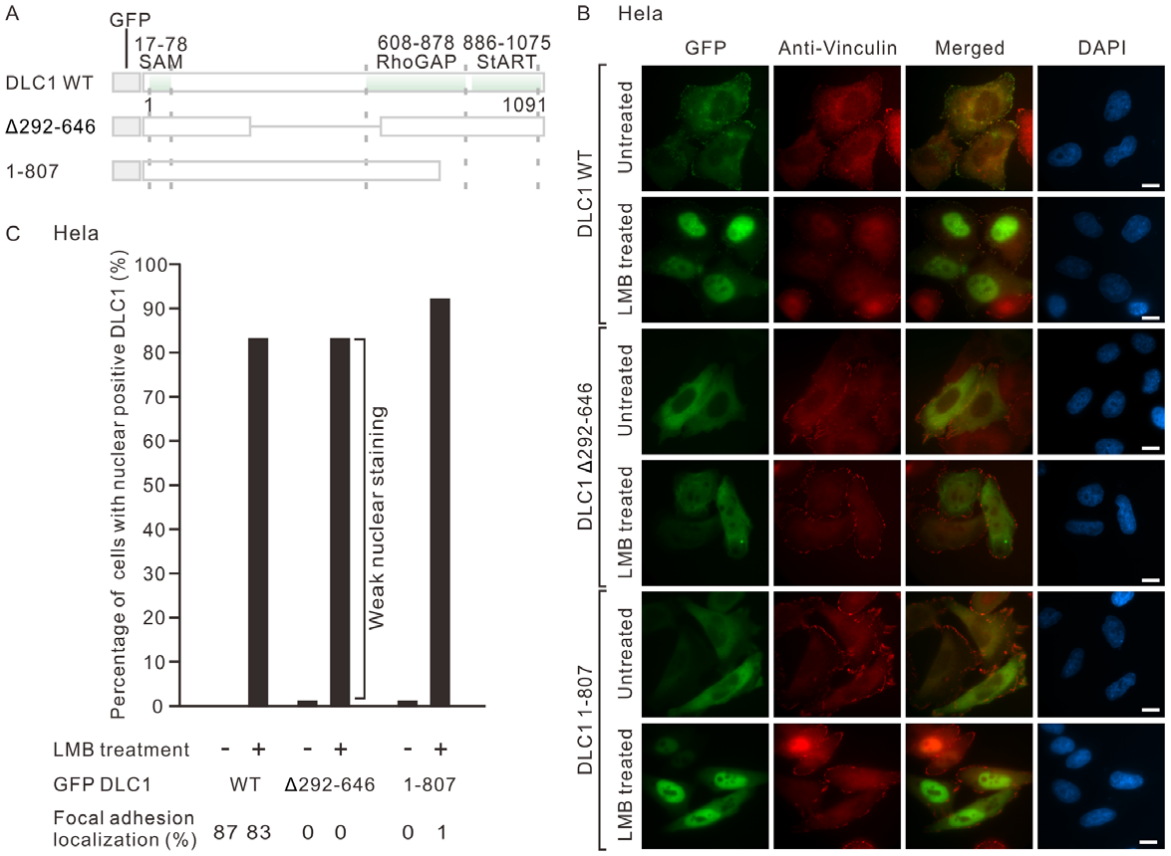
Center region of DLC1 was important for its nuclear localization. (**A**) Schematic diagram showing the structure of wildtype DLC1 (DLC1 WT) and its deletion mutants (Δ292–646 and 1–807). (**B**) HeLa cells were transiently transfected with the GFP-tagged DLC1 expression constructs as listed in (A), followed by LMB treatment. Focal adhesions were counterstained with anti-vinculin antibody. The subcellular localization of DLC1 was recorded by counting at least 100 transfected cells per sample. (**C**) Bar graph summarizing the subcellular localization of DLC1 and its mutants in (B) with or without the LMB treatment. The results represent a duplicate of two independent experiments. The percentages of focal adhesion positive DLC1 in individual group were also highlighted.

We questioned whether, apart from the nuclear targeting motif suggested by the others, additional region in DLC1 is involved in the nuclear localization of DLC1. To address this, we cloned and expressed GFP-DLC1 deletion mutants and examined their localization after LMB treatment in HeLa cells (**Fig. 2A**). We found that DLC1 Δ292–646 internal deletion mutant showed a significant reduction of nuclear localization upon LMB treatment (**Fig. 2B and 2C**). On the other hand, nuclear retention of DLC1 1–807 C-terminal deletion mutant was not affected when compared with wildtype DLC1 upon LMB treatment. Interestingly, 1–807 mutant which contained the proposed focal adhesion targeting region (region 200–500) [18] could not be efficiently targeted to focal adhesions and diffusely expressed in the cytoplasm.

It has been proposed that the N-terminal region of DLC1 negatively regulates its nuclear entry [16]. A number of potential nuclear export signal (NES) has been mapped at 62–71, 764–773 and 792–801 residues of DLC1 by *in silico* search (**Fig. S2A**). The importance of these signal sequences in regulating the cytoplasmic localization was then assessed by immunofluorescence. The functionality of NES at 764–773 and 792–801 residues were excluded by the cytoplasmic expression of 1–291 and 1–400 mutants (**Fig. S2B**). Since 609-stop and 648–839 mutants displayed enhanced nuclear localization, the role of NES at 62–71 residues was further analyzed by creating a DLC1 Δ62–71 mutant. This DLC1 mutant showed characteristic focal adhesion localization similar to the wild-type, without showing any increase in nuclear retention (**Fig. S2B**). To clarify the absence of nuclear retention was not due to the strong focal adhesion association, DLC1 1–807Δ62–71, a mutant failed to localize at focal adhesions was expressed. However, this mutant was also found to be predominantly localized at the cytoplasm. Taken together, we found that removal of the predicted NES at the N-terminus of DLC1 did not affect its nucleocytoplasmic distribution.

**Figure 3:**
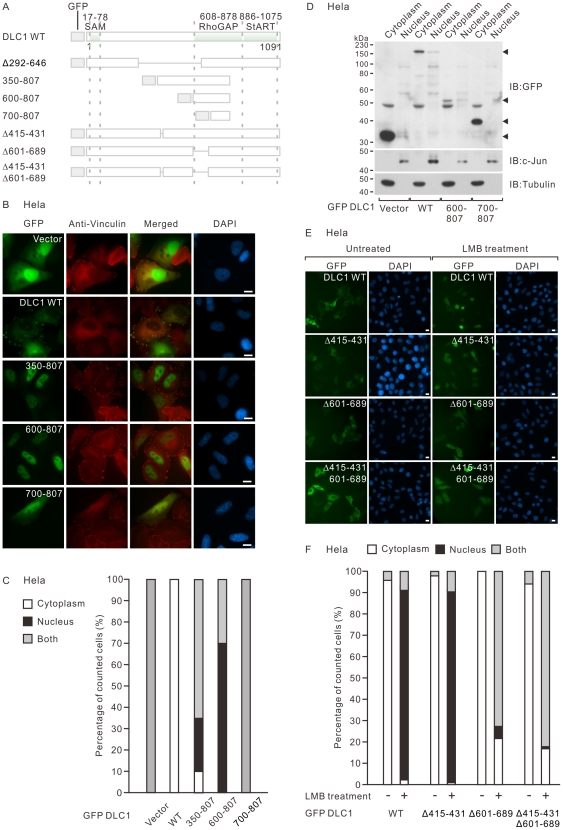
DLC1 600–807 within the center region was important for its nuclear localization. (**A**) Schematic diagram showing the structure of a panel of DLC1 fragments terminated at amino acid 807 and a series of DLC1 internal deletion mutants for mapping the key nuclear targeting site. The structures of wildtype DLC1 and Δ292–646 were also listed for comparison. (**B**) HeLa cells were transiently transfected with the GFP-tagged DLC1 expression constructs indicated. Focal adhesions were counterstained with anti-vinculin antibody. The subcellular localization of DLC1 was recorded by counting at least 100 transfected cells per sample. (**C**) Bar graph summarizes the percentage of cells with cytoplasmic and nuclear localization of DLC1 and its mutants in (B). The results represent a duplicate of two independent experiments. (**D**) HeLa cells were transiently transfected with the indicated GFP-tagged DLC1 expression constructs followed by subcellular fractionation. The fractionated cytoplasmic and nuclear lysates were subjected to immunoblotting with antibodies against GFP, tubulin (cytoplasmic marker) and c-jun (nuclear marker). (**E**) HeLa cells were transiently transfected with the indicated GFP-tagged DLC1 expression constructs, followed by LMB treatment. (**E**) Bar graph summarizes the percentage of cells with cytoplasmic and nuclear localization of DLC1 and its mutants in (D). The results represent a duplicate of two independent experiments. Scale bar: 10 µm.

After highlighting the nuclear targeting region to the center region of DLC1, we performed the first detailed localization analysis of a panel of GFP-DLC1 mutants (**Fig. 3A**). Interestingly, we found that expression of 350–807 mutant showed a predominant nuclear localization. Similar prominent nuclear localization was seen in Myc-DLC1 291–807 (data not shown). Examination of 600–807 mutant revealed similar nuclear localization, while 700–807 mutant localized in both cytoplasm and nucleus in HeLa cells (**Fig. 3B and 3C**). Increased nuclear localization of 600–807 mutant was also supported by the strongest DLC1 expression in the nuclear fraction obtained in the biochemical fractionation assay when compared with the other DLC1 mutants (**Fig. 3D**). Taken together, we found that DLC1 region 600–700 in the RhoGAP domain was also involved in the nuclear entry of DLC1.

To further confirm the importance of this novel region, we deleted region 601–689 in DLC1 and assessed its localization. Interestingly, we found a marked reduction in the nuclear localization of DLC1Δ601–689 mutant after LMB treatment (**Fig. 3E and 3F**). As demonstrated by the Δ415–431Δ601–689 mutant in which the previously reported NLS and our mapped site were deleted, deletion of 415–431 did not further reduced the nuclear retention much after LMB treatment. Our results suggested that the mapped novel site plays a significant role in targeting DLC1 into the nucleus.

### Nuclear DLC1 lost its inhibitory activity in suppressing colony formation and actin stress fiber formation *in vitro*

**Figure 4:**
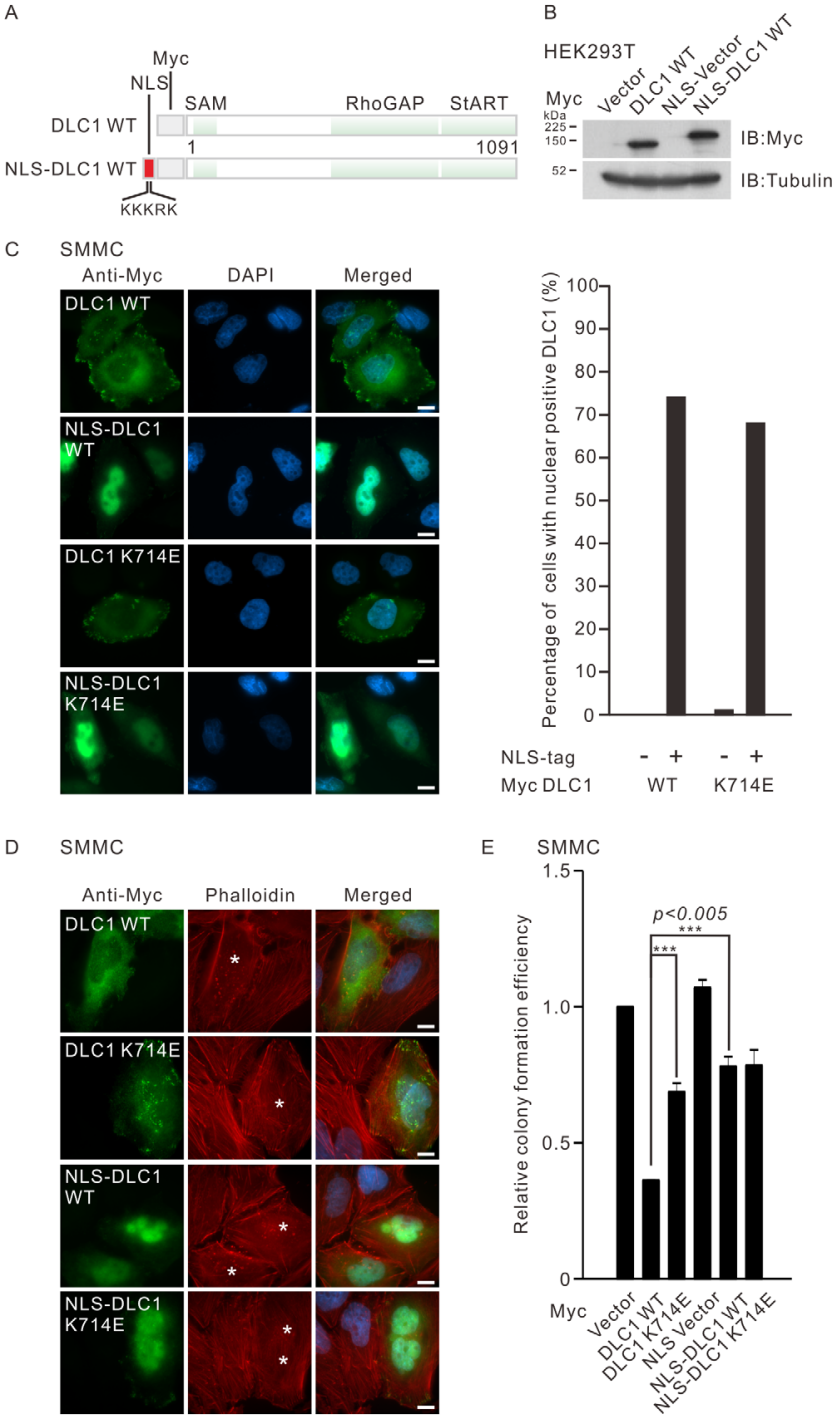
Transiently expressed nuclear-targeted DLC1 exhibited reduced activity in suppressing actin fiber formation and colony formation. (**A**) Schematic diagram showing the structure of the nuclear targeted DLC1 (NLS-DLC1) used for transient expression experiment. The DNA sequence encoding the monopartite NLS which consists of five basic amino acids KKKRK were inserted in front of the open reading frame of Myc-tagged DLC1. Translation of the manipulated expression construct encoded Myc-tagged NLS-DLC1. (**B**) HEK293T lysates with overexpressed Myc-tagged DLC1 or NLS-DLC1 were immunoblotted with anti-Myc antibody. (**C**) SMMC-7721 cells were transiently transfected with the indicated wildtype DLC1 or the RhoGAP mutant (K714E) constructs. The Myc-tagged protein was visualized by anti-Myc antibody followed by FITC-conjugated secondary antibody. The subcellular localization of DLC1 was recorded by counting at least 100 transfected cells per each sample. Bar graph indicates the percentage of cells with nuclear DLC1 staining. The results represent a duplicate of two independent experiments. (**D**) SMMC-7721 cells were transiently transfected with the indicated Myc-tagged DLC1 constructs and the actin stress fibers were stained with TRITC-conjugated phalloidin 1 hour after the serum induction. The asterisk (*) marks the DLC1-transfected cells. (**E**) Bar graph showing the relative colony formation efficiency of SMMC-7721 cells being transfected with the indicated DLC1 constructs and pEGFP-C1 vector carrying neomycin resistant gene in 10∶1 ratio. The transfected cells were selected with G418 for 2 weeks. The colonies formed were visualized by crystal violet staining and quantified. The mean difference between the indicated group was found to be statistically significant (***P<0.005; unpaired *t*-test). Scale bar: 10 µm.

To date, there is no study assessing the direct functional association between nuclear localization and the biological function of DLC1. To measure the functional capacity of nuclear DLC1 *in vitro*, we created and confirmed the expression of a nuclear targeted DLC1 (NLS-DLC1) (**Fig. 4A and 4B**). By immunofluorescence, we confirmed that NLS-DLC1 was predominantly localized in the nucleus in SMMC-7721 cells. An NLS-driven DLC1 RhoGAP mutant, NLS-K714E could also be targeted to the nucleus as efficiently as the wildtype DLC1 (**Fig. 4C**). We then examined the integrity of actin stress fibers in SMMC-7721 cells transiently transfected by these mutants. Unlike the disassembly of the actin stress fibers in the DLC1 transfected cells, NLS-DLC1 could only partially suppress the actin stress fiber formation (**Fig. 4D**). As a negative control, cells transfected with DLC1 K714E displayed intact actin stress fibers. Similar pattern was observed in cells expressing NLS-K714E further supporting that our nuclear targeting system did not exert nonspecific effect on actin stress fiber formation. RhoGAP activity of DLC1 has been shown to be tightly associated with its growth suppressive activity. We then performed colony formation assay to assess the *in vitro* growth suppressive activity of NLS-DLC1 in both SMMC-7721 and HeLa cells (data not shown). In accordance with the effect on stress fiber formation, NLS-DLC1 showed largely reduced growth suppressive activity when compared with DLC1 (**Fig. 4E**) Taking together, we found that nuclear DLC1 lost capacity to suppress cell growth.

### Nuclear DLC1 did not suppress *in vivo* tumorigenicity

**Figure 5:**
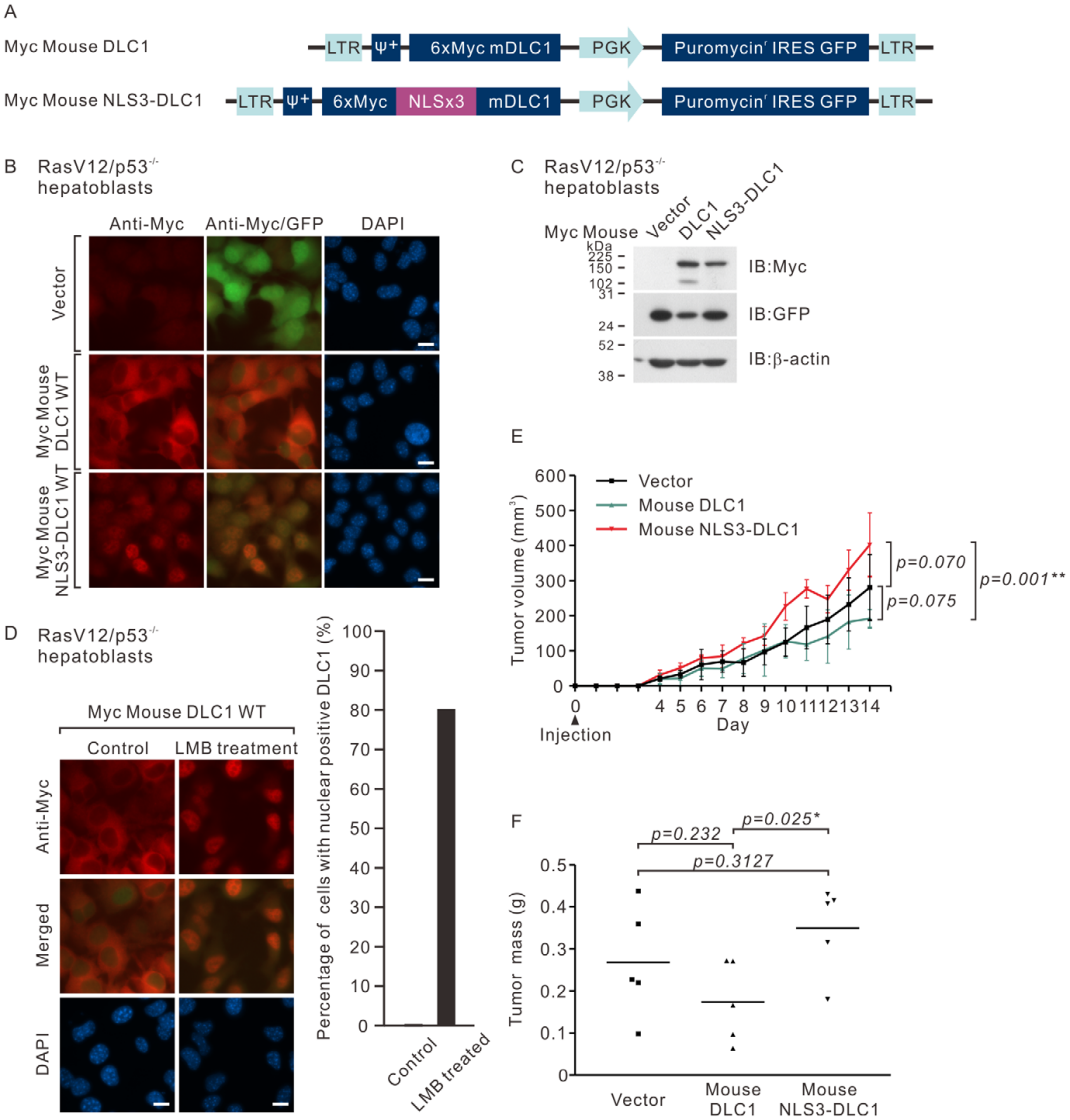
Mouse RasV12/p53^−/−^ hepatoblasts with stable nuclear targeted DLC1 expression exhibited reduced tumor suppressive activity *in vivo.* (**A**) Schematic diagram illustrating the retroviral vectors used to drive the stable expression of Myc-tagged mouse DLC1 and the nuclear-targeted mouse DLC1 (NLS3-DLC1). (**B**) Hepatoblasts infected with vector, DLC1 or NLS3-DLC1 retroviruses were fixed and the expression of Myc-tagged protein was visualized by anti-Myc antibody followed by Texas Red-conjugated secondary antibody. (**C**) Lysates of infected hepatoblasts with vector, DLC1 or NLS3-DLC1 retroviruses were subjected to immunoblotting using antibodies against Myc, DLC1 and GFP. β-actin was served as the loading control. (**D**) Hepatoblasts infected with DLC1 retrovirus were subjected to LMB treatment. The cells were then fixed and the localization of the Myc-tagged DLC1 was visualized by anti-Myc antibody followed by Texas Red-conjugated secondary antibody. Bar graph indicates the percentage of cells with nuclear DLC1 staining. The results represent a duplicate of two independent experiments. (**E**) The tumorigenicity of the indicated retroviral transduced hepatoblasts was assessed by subcutaneous nude mice injection. 1×10^5^ hepatoblasts were subcutaneously injected at day 0 and the volume of the tumor size was monitored daily from day 4. All mice were sacrificed at day 14. At day 14, the mice were sacrificed and the tumors were dissected. (**F**) The dissected tumors were weighed and their masses were recorded. Scale bar: 10 µm.

To assess the tumor suppressive activity of nuclear DLC1 *in vivo*, we performed stable retroviral expression of nuclear targeted Myc-tagged mouse DLC1 in a p53 null mouse hepatoblasts with constitutive RasV12 activation [11]. DLC1 was targeted to the nucleus by inserting a tandem repeat of three monopartite NLSs (NLS3) in front of the start codon of mouse DLC1 in the retroviral expression construct (**Fig. 5A**). After selection with puromycin, the resistant clones were expanded and over 95% resistant clones were found to be GFP positive, confirming the high retroviral transduction efficiency (**Fig. 5B**). We also confirmed the successful nuclear targeting of mouse DLC1 by immunofluorescence staining for NLS3-DLC1 fusion protein. We found that over 90% of the NLS3-DLC1 transduced cells showed nuclear staining when compared with the cytoplasmic staining in DLC1 transduced cells. Background staining with anti-Myc antibody was barely detectable in the vector cells (**Fig. 5B**). We further confirmed the expression level DLC1 proteins by immunoblotting (**Fig. 5C**). To further confirm that the property of nucleocytoplasmic shuttling was conserved in our DLC1 overexpressing hepatoblast model, we treated DLC1 expressing cells with LMB, followed by immunofluorescence. We found that treatment with LMB could consistently retain DLC1 in the nucleus (**Fig. 5D**). This observation further supported that the nucleocytoplasmic shuttling property was conserved in the human and mouse DLC1 orthologs. The *in vivo* tumorigenicity of these stable clones was examined by subcutaneous injection in the nude mice. Consistently, DLC1 exhibited suppression effect on tumor formation, whereas nuclear DLC1 showed a significant reduction in suppressing tumorigenicity *in vivo* (**Fig. 5E and 5F**).

It has been reported that ectopic expression of DLC1 in DLC1 negative cells could induce apoptosis [19]. To test whether NLS3-DLC1 exhibited differential biological activity in inducing apoptosis, we established and analyzed HEK293T cells stably expressing nuclear DLC1 by flow cytometry for the subG1 cell population (**Fig. S3B**). To avoid any possible clonal effect, two clones from each group were picked for the analysis. We found that DLC1 expressing cells showed an increased subG1 peak when compared with the vector and the NLS3-DLC1 expressing cells. DLC1 expressing cells also showed a reduced G1 but increased S population (**Fig. S3B**). Plausible explanation to this could be that DLC1 expressing cells may spend longer time in S phase once they enter the cell cycle. These observations again demonstrated that NLS3-DLC1 was less robust in inducing apoptosis and attenuating cell cycle progression when compared with the wildtype DLC1.

## Discussion

*In silico* sequence analysis has revealed a number of potential basic amino acid rich bipartite NLSs in DLC1 [16]. The presence of these motifs prompted us to ask whether DLC1 exists as a nuclear protein. In this study, we demonstrated that DLC1 was a protein continuously shuttled between cytoplasm and nucleus in different cell lines. This observation provides a balanced explanation for the presence of nuclear DLC1 and supports its intrinsic cytoplasmic localization as demonstrated by different studies [10], [18], [20], [21], [22]. It is noteworthy that upon the treatment with LMB, focal adhesion localized DLC1 was still detectable. This observation suggested that only non-specific, cytoplasmic DLC1 was retained in the nucleus after the treatment. Focal adhesion localized DLC1 was relatively static in nature. This observation is compatible to the partial nuclear staining observed for the DLC1 focal adhesion localization defective mutant, Y442F in a lung cell model [10]. Although positive nuclear staining could be found in normal non-neoplastic liver in immunohistochemical staining, it would be interesting to observe whether there is a distinguishable difference of nuclear DLC1 staining between the normal liver tissue and DLC1-positive HCCs (or other cancer types).

Although the presence of nuclear DLC1 has been previously discussed, its tumor suppressive activity in the nucleus has not been clearly addressed. To directly investigate the tumor suppressive activity of the nuclear DLC1, we transiently or stably expressed NLS-DLC1 in HCC cell lines followed by a series of functional characterization. We used this approach rather than studying the DLC1 mutant lacking the mapped nuclear targeting signal (600–700 residues were overlapped with the RhoGAP domain) as we predicted that removing this signal would disrupt its intrinsic RhoGAP activity and make the comparison with the wildtype DLC1 impossible. Also, long term LMB treatment to produce nuclear DLC1 is also unfavorable and it blocks global nuclear protein export and poses stress to the cells [23]. While the novel function of nuclear DLC1 remains to be explored, we confirmed that NLS-DLC1 was less effective in suppressing cell growth, disintegrating RhoA-mediated actin stress fiber formation, and inducing cell death *in vitro*. NLS-DLC1 was also less effective in suppressing tumor growth in a mouse xenograft model. We speculate nuclear mislocalization may be a potential mechanism in abrogating DLC1 activity in human cancer with intact DLC1 expression.

Functionally, our findings are inconsistent with the conclusion made by previous reports. Yuan et al. demonstrated nuclear translocation of DLC1 can induce apoptosis and facilitate its tumor suppressive function in a transiently expressed DLC1 negative human non-small cell lung cancer (NSCLC) cell line model [16]. However, our stable nuclear DLC1 expression model with mouse hepatoblasts and human HEK293T cells neither failed in enhancing nor inducing apoptotic sub-population as indicated by flow cytometry analysis. Plausible explanation includes transient expression produces a very high level of DLC1 that may cause toxicity to the cells; while our stable cell system may have undergone some kind of adaptation during the stable cell selection, which allows the cells to be propagated with the expression of nuclear DLC1. Also, the cell type and cell line dependent effects in contributing to the difference in DLC1 activity of inducing apoptosis and growth arrest have to be considered [11], [16], [24]. On the other hand, Scholz et al has shown that DLC1 S327AS431A mutant is unable to complex with 14-3-3 in the cytoplasm and is biologically more active than wildtype DLC1. Unfortunately, the authors found that introduction of negatively charged residues at the aforementioned sites cannot mimic the constitutively active 14-3-3 binding conformation. The lack of a favorable mutant hinders the assessment on how these 14-3-3 binding residues affect the LMB sensitivity of DLC1 [17]. By assuming the DLC1 S327AS431A is less likely to be sequestered in the cytoplasm, one may predict that this mutant can be shuttled into the nucleus more often. However, it is currently unknown whether the hyperactivity of this mutant is a result of the increased nuclear entry in short term or is due to the conformational change that favors DLC1 RhoGAP function. It is possible that the increased nuclear entry of a biological hyperactive DLC1 may serve as a negative regulatory mechanism to downregulate the hyperactive DLC1 in a long term.

Two independent studies have attempted to map the NLS in DLC1 [16], [17]. Yuan et al suggested the DLC1 415–431 is the putative NLS, while Scholz et al suggested that DLC1 nuclear transport requires only the latter half of the same site involving the residues 428 and 429. However, we found that DLC1 mutant lacking the whole proposed NLS could still be effectively retained in the nucleus, indicating extra structural sequence in DLC1 may be involved in this nuclear transport. To address this, we performed detailed subcellular localization examination of GFP-DLC1 deletion mutants. We found that DLC1 Δ292–646 mutant was less LMB sensitive. Consistently, we found that expression of fragments representing only the center region of DLC1 showed nuclear localization. The observation implies that alteration at both ends of DLC1 may expose elements which are driving nuclear localization of DLC1. From these data, we further propose and confirm that the region 600–700 in the RhoGAP domain is important in directing nuclear localization of DLC1. LMB is a global nuclear export blocker which, non-specifically blocks numerous protein export besides DLC1. It remains to be tested whether the mapped site is functionally independently; or is capable to interact with other biomolecules in regulating the nuclear import of DLC1. It has been suggested that the SH3 domain of p120RasGAP interacts with the DLC1 RhoGAP domain and in turn inactivates its activity [25]. Also, the phospholipid PIP3 has also been proposed to be required for the optimal activity of DLC1 through the interaction of the polybasic region resides in the RhoGAP domain [26]. Further investigation of the involvement of other proteins and lipids by using DLC1 mutants with key interacting residues being mutated as well as cell model with well defined genetic background is definitely useful to answer the mechanistic regulation regarding DLC1 nuclear entry. In addition, in an attempt to map the potential NES at 62–71 residues of DLC1, we could not observe a significant change in the localization of DLC1 mutant without this potential NES.

There are some issues regarding the nuclear DLC1 that remain to be resolved. It is important to determine the extracellular stimulus and the intracellular signaling pathways guiding the nucleocytoplasmic shuttling of DLC1. Currently, DLC1 had been suggested to be a potential substrate of Akt, p70S6-kinase [27] and protein kinase D (PKD) [17] pathways. Our group has recently shown that Akt phosphorylates DLC1 at S567 residue and inactivates its tumor suppressive activity through a RhoGAP independent mechanism [28]. However, this particular Akt phosphorylation did not seem to alter focal adhesion localization and nucleocytoplasmic shuttling activity of DLC1. (unpublished observations). Scholz et al suggests that PKD-mediated DLC1 phosphorylation at S327 and S431 residues can facilitate DLC1-14-3-3 interaction, followed by masking the potential NLS and sequestrating DLC1 in the cytosol in breast cancer cells [17]. However, we could not observe significant difference in terms of the efficiency of nuclear entry among the wildtype DLC1, S431A and S431D in LMB-treated SMMC-7721 cells (unpublished observations).

Besides acting as a potential reservoir in the spatial regulation of the tumor suppressive activity of DLC1, it is tempting to investigate the nuclear functions of DLC1. It is known that DLC1 inactivates Rho and interacts with tensin proteins at focal adhesions in the cytoplasm; however, it is not known whether DLC1 will interact with a nuclear specific subset of substrates or coupled with its known cytoplasmic substrate into the nucleus. Recently, Dubash et al further demonstrates nuclear Net1 can activate nuclear fraction of RhoA in responding to ionizing radiation induced DNA damage response in a HEK293 cell model. Their findings further indicate that spatial distribution of Rho signaling regulators may pose an extra layer of complexity to the RhoA signaling network in responding to different biological stimuli [29]. Follow up study to explore the novel function of nuclear DLC1 will definitely provide new insight about the functions as well as the general understanding about the biological regulation of RhoGTPase signaling.

## Materials and Methods

### Ethics statement

The use of animal model in this study was approved by the Committee on the Use of Live Animals in Teaching and Research (CULATR), The University of Hong Kong. All the protocols were strictly performed under the approved research protocol (CULATR 1977-09) and the Animals (Control of Experiments) Ordinance (Hong Kong).

### Cell culture and transfection

Human hepatoma cell lines, HepG2, Hep3B and SK-Hep1; human cervical carcinoma cell line HeLa and human embryonic kidney cell line HEK293T were obtained from American Type Culture Collection (Manassas, VA, USA), whereas the human HCC cell lines, SMMC-7721 and BEL7402 were obtained from the Shanghai Institute of Cell Biology. Mouse p53^−/−^; RasV12 hepatoblastoma cell line was a generous gift from Dr. Scott W. Lowe of Cold Spring Harbor Laboratory, USA. HepG2, HEK293T, HeLa, SMMC-7721, BEL7402 and p53^−/−^; RasV12 cells were maintained in DMEM high glucose medium supplemented with 10% (v/v) fetal bovine serum, penicillin and streptomycin. SK-Hep1 and Hep3B cells were maintained in MEM medium supplemented with 10% (v/v) fetal bovine serum, penicillin and streptomycin. All cells were cultured in a humidified incubator at 37°C with 5% CO_2_ in air. Transfection with the indicated plasmid was done with Lipofectamine 2000 reagent, according to the manufacturer's instructions (Invitrogen, Carlsbad, CA, USA).

### Plasmids

DNA expression constructs using pCS2+MT, pEGFP-C1 (BD Biosciences Clontech, Palo Alto, CA, USA) and pMSCV-6X-Myc-Puro-IRES-GFP (gift from S. W. Lowe) vectors were prepared by standard molecular cloning techniques and PCR amplification of the described fragments. Eukaryotic expression vectors for Myc-tagged proteins were derived from pCS2+MT and prepared as follows: DLC1 1–1091 (WT, wildtype), K714E (RhoGAP mutant) and Δ415–431. NLS-DLC1 and NLS K714E were prepared by subcloning the DLC1 WT and K714E cDNAs into the pCS2+MT derived pCS2+MT+NLS vector. In brief, a pair of complementary oligonucleotides 5′-GATCCGCCGCCATGGCTCCAAAGAAGAAGCGTAA GGTAAAT-3′ and 5′-CGATTTACCTTACGCTTCTTCTTTGGAGCCATGGCGGCG-3′ was reannealed, double digested with BamHI and ClaI, followed by subcloning into the multiple cloning sites of the pCS2+MT vector. Eukaryotic expression vectors for GFP-tagged proteins were derived from pEGFP-C1 and prepared as follows: DLC1 WT, Δ292–646, Δ62–71, Δ415–431, Δ601–689, Δ415–431Δ601–689, 1–291, 1–400, 1–807, 1–807 Δ62–71, 350–807, 600–807, 700–807, 609-stop, 648–839. Retroviral expression vectors for Myc-tagged proteins were derived from pMSCV-6X-Myc-Puro-IRES-GFP and prepared as follows: mouse DLC1 WT (gift from S. Lowe). Mouse NLS-DLC1 was prepared by first ligating BamHI cut cDNA of a tandem repeat of three basic, monopartite NLSs in front of the multiple cloning site of the retroviral expression vector, followed by the subcloning of the mouse DLC1 cDNA in frame behind the 3′ region of the inserted NLSs. All DNA expression constructs were confirmed by DNA sequencing.

### Immunofluorescence microscopy and LMB treatment

For the subcellular localization studies, cells were fixed with 4% paraformaldehyde in PBS for 15 minutes and then permeabilized with 0.2% Triton-X-100 in PBS for 10 minutes, followed by blocking with 3% bovine serum albumin in PBS for 20 minutes at room temperature. The blocked coverslips were then incubated with the primary antibody, followed by FITC- or Texas Red-conjugated secondary antibody, for 1 hour each. Myc-tagged protein was stained with monoclonal anti-Myc antibody (Santa Cruz Biotechnology, Santa Cruz, CA, USA). Endogenous DLC1 was stained with polyclonal anti-DLC1 antibody (Santa Cruz Biotechnology). The endogenous vinculin was stained by monoclonal anti-vinculin (Sigma, St. Louis, MO, USA) antibody. The F-actin was visualized by tetramethylrhodamine B isothiocyanate (TRITC)-labeled phalloidin (Sigma) one hour after serum induction. The processed coverslips were mounted in Vectashield anti-fade mounting medium (Vector Laboratories, Burlingame, CA, USA). Images were captured by a Leica Q550CW fluorescence microscope (Leica, Wetzler, Germany).

For the LMB (Sigma) treatment, a protocol of 10 ng/ml LMB for 6 hours was employed, unless stated otherwise in specific experiment. To study the effect of LMB on transiently expressed protein, cells were transfected with the indicated DLC1 construct for 16 hours before the treatment. For the study concerning the endogenous DLC1, LMB treatment was employed 24 hours after seeding cells on the coverslips.

### Subcellular fractionation

To harvest cells for fractionation, 2×10^6^ cells were washed once with PBS and harvested by trypsinization followed by centrifugation at 1500×g for 5 minutes. The cell pellet was further washed with 10 ml PBS and was then transferred to a microcentrifuge tube. Cell pellet was collected by centrifugation at full speed for 15 seconds. To perform cell lysis and extract the cytoplasmic fraction, cell pellet was gently resuspended in 400 µl ice-cold buffer A (10 mM HEPES, pH7.9, 10 mM KCl, 0.1 mM EDTA, 0.1 mM EGTA, 1 mM DTT and 0.5 mM PMSF) and was incubated on ice for 15 minutes. Twenty percent NP-40 (v/v) solution of 12.5 µl was then added to the cell suspension followed by vigorous vortexing for 10 seconds. The homogenate was cleared by centrifugation at full speed for 30 seconds. The supernatant was collected as the cytoplasmic fraction. To extract the nuclear fraction, the nuclear pellet was resuspended in 50 µl ice-cold buffer C (20 mM HEPES, pH7.9, 400 mM NaCl, 1 mM EDTA, 1 mM EGTA, 1 mM DTT and 1 mM PMSF) and was incubated on ice for 15 minutes with vortex every 3 minutes. The nuclear extract was collected by cold centrifugation at full speed for 5 minutes. The supernatant contained the nuclear fraction. Ten to twenty µg of extract from each fraction was separated on a 10% SDS-PAGE. The endogenous tubulin and c-Jun was immunoblotted with monoclonal anti-tubulin (Sigma) and anti-c-Jun antibodies (BD-transduction, San Jose, CA, USA) as cytoplasmic and nuclear markers respectively.

### Cell lysis and western blotting

The harvested cells were lysed with NET-N buffer (25 mM Tris-HCl, pH 8.0, 50 mM NaCl, 0.2 mM EDTA, 0.1% NP40) and a cocktail of protease inhibitors (Roche, Mannheim, Germany) on ice for 20 minutes. The cell lysate was cleared by centrifugation at 16,000×g for 15 minutes at 4°C. Ten to twenty µg of boiled cell lysates were subjected to SDS-PAGE analysis. The Myc-tagged and GFP-tagged proteins were immunoblotted with monoclonal anti-Myc and anti-GFP antibodies (Santa Cruz Biotechnology). The endogenous DLC1 was immunoblotted with polyclonal anti-DLC1 antibody (Santa Cruz Biotechnology). Endogenous tubulin or β-actin was detected by monoclonal anti-tubulin or anti-β-actin antibodies (Sigma) as a loading control.

### Immunohistochemistry

Immunohistochemical staining for DLC1 was performed on formalin-fixed, paraffin-embedded sections using the EnVision™ + Kits (Dako, Glostrup, Denmark). The cut sections were first dewaxed in xylene, rehydrated through graded ethanol. For antibody retrieval, the sections were boiled with EDTA (1 mM, pH = 8.0) solution for 15 minutes. To block the endogenous peroxidase activity, the sections were treated for 3% H_2_O_2_ for 30 minutes. The samples were then incubated with rabbit polyclonal anti-DLC1 antibody for 1 hour at room temperature. The stained protein was visualized using EnVision™+Kits (Dako) according to the suggested protocol by the manufacturer. Nucleus was counterstained by hematoxylin. The stained sections were dehydrated, mounted on glass slides and observed under the microscope.

### Colony formation assay

Cells were seeded at 1×10^5^ cells per well on a 6-well plate were transfected with Myc-tagged DLC1 construct and pEGFP-C1 vector carrying neomycin resistant gene in 10∶1 ratio for 24 hours. Transfected cells were then seeded with medium containing 0.5 mg/ml G418 for two weeks. The colonies formed were fixed after selection and visualized by crystal violet staining. The number of colonies was analyzed by AlphaEase FC Software (Alpha Innotech Corporation).

### Retroviral packaging and transduction

For transient retroviral packaging, pMSCV-Puro-IRES-GFP based retroviral construct was transfected into PA317 packaging cells with Lipofectamine 2000 reagent. Sixteen hours after transfection, cells were cultured in 10 ml fresh medium to collect the retroviral particles for 24 hours. To purify the retroviral particles, the collected medium was transferred to a clean centrifuge tube and the cell debris was cleared by centrifugation at 1667×g for 3 minutes at room temperature. The supernatant was then used for subsequent viral transduction.

For stable retroviral transduction, 5×10^4^ p53^−/−^; RasV12 hepatoblasts or HEK293T cells were seeded on a six well plate one day before viral transduction. The cells were incubated with 1 ml viral particles supplemented with 8 µg/ml polybrene for 24 hours, followed by a recovery period of 48 hours in fresh medium. The viral-infected hepatoblasts and HEK293T cells were then selected with 5 µg/ml and 0.5 µg/ml puromycin respectively for 10 days.

### Subcutaneous Injection in Nude Mice

To assess the tumorigenicity of mouse DLC1 overexpressing hepatoblasts, 1×10^5^ cells suspended in 100 µl PBS were injected subcutaneously into the back of male BALB/C nude mice at 4 weeks of age. Five mice were injected per stable clone. Tumor sizes were measured daily starting at day 4 post-injection. Mice were sacrificed at day14 post-injection and the tumors were then excised, weighed and photographed. Tumor volume was calculated by the formula: ½×L×W^2^ (L: length of tumor; W: width of tumor). This experiment was performed according to the Animals (Control of Experiments) Ordinance (Hong Kong) and all the procedures were strictly followed the guidance on animal experimentation set out by the University of Hong Kong.

### Propidium iodide staining and flow cytometry

To prepare cells for propidium iodide staining, 5×10^5^ HEK293T cells were harvested by trypsinization and were fixed with ice-cold 80% ethanol in PBS. The fixed cells were then treated with 50 µg/ml RNaseA for 30 minutes at 37°C followed by DNA staining with 50 µg/ml propidium iodide for 5 minutes at room temperature. The DNA content of the cells was then analyzed by FACSCalibur flow cytometer (BD Biosciences).

## Supporting Information

Figure S1 **DLC1 ΔNLS (Δ415–431) could still be retained in the nucleus upon LMB treatment in SMMC–7721 cells.** (**A**) Schematic diagram showing the proposed NLS in DLC1 as stated by Yuan et al and Schloz et al. The S431 site was proposed as a 14-3-3 docking site which modulates the DLC1 nuclear entry by Scholz et al. The structure of DLC1 ΔNLS (Δ415–431) and S431A mutants were outlined. (**B**) Western blotting showing the protein expression of Myc-tagged wild type DLC1 and ΔNLS mutant in transiently transfected SMMC-7721 cells. (**C**) Immunofluorescence staining showing the localization of wildtype DLC1 and ΔNLS in the presence or absence of LMB. DLC1 was visualized with anti-Myc antibody following by FITC conjugated antibody. Nucleus was counterstained with DAPI. The subcellular localization of DLC1 was recorded by counting at least 100 transfected cells per sample. Bar graph summarizing the subcellular localization patterns of DLC1 and the ΔNLS mutant. The results represent a duplicate of two independent experiments. (**D**) Immunofluorescence staining showing the localization of wildtype DLC1 and S431A mutant in cells in the presence or absence of LMB. Bar graph summarizing the subcellular localization patterns of DLC1 and the ΔNLS mutant. The results represent a triplicate of three independent experiments. Scale bar: 10 µm.
(TIF)

Figure S2 **Prediction and characterization of potential Nuclear Exporting Signals (NES) in DLC1.** (**A**) Schematic diagram showing the position of the three potential NESs in DLC1 based on the *in silico* search for Leucine rich motif (LXXXLXXLXX; L = Leucine; X = Any amino acids). The structure and subcellular localization of DLC1 constructs used to pinpoint the potential NES were listed. (B) (**E**) HeLa cells were transiently transfected with GFP-tagged DLC1 expression constructs listed in (A). Focal adhesions were counterstained with anti-vinculin antibody. Scale bar: 10 µm.
(TIF)

Figure S3 **Stable expression of nuclear targeted DLC1 was less potent in inducing apoptotic subG1 cell population in HEK293T cells.** (**A**) HEK293T cells were transduced with the indicated retroviruses. Two individual clones of each group were picked and propagated. Transduced cells were GFP positive. HEK293T cells transduced with the indicated retroviruses were lysed and subjected to immunoblotting using anti-Myc and anti-GFP antibodies. Tubulin was served as the loading control. (**B**) HEK293T cells transduced with the indicated retroviruses were subjected to flow cytometry analysis for propidium iodide staining. The cell cycle profiles of individual cell lines were shown.
(TIF)

## References

[1] Yuan BZ, Miller MJ, Keck CL, Zimonjic DB, Thorgeirsson SS (1998). Cloning, characterization, and chromosomal localization of a gene frequently deleted in human liver cancer (DLC-1) homologous to rat RhoGAP.. Cancer Res.

[2] Ng IO, Liang ZD, Cao L, Lee TK (2000). DLC-1 is deleted in primary hepatocellular carcinoma and exerts inhibitory effects on the proliferation of hepatoma cell lines with deleted DLC-1.. Cancer Res.

[3] Kim TY, Jong HS, Song SH, Dimtchev A, Jeong SJ (2003). Transcriptional silencing of the DLC-1 tumor suppressor gene by epigenetic mechanism in gastric cancer cells.. Oncogene.

[4] Yuan BZ, Zhou X, Durkin ME, Zimonjic DB, Gumundsdottir K (2003). DLC-1 gene inhibits human breast cancer cell growth and in vivo tumorigenicity.. Oncogene.

[5] Yuan BZ, Jefferson AM, Baldwin KT, Thorgeirsson SS, Popescu NC (2004). DLC-1 operates as a tumor suppressor gene in human non-small cell lung carcinomas.. Oncogene.

[6] Guan M, Zhou X, Soulitzis N, Spandidos DA, Popescu NC (2006). Aberrant methylation and deacetylation of deleted in liver cancer-1 gene in prostate cancer: potential clinical applications.. Clin Cancer Res.

[7] Ullmannova V, Popescu NC (2006). Expression profile of the tumor suppressor genes DLC-1 and DLC-2 in solid tumors.. Int J Oncol.

[8] Wong CM, Yam JW, Ching YP, Yau TO, Leung TH (2005). Rho GTPase-activating protein deleted in liver cancer suppresses cell proliferation and invasion in hepatocellular carcinoma.. Cancer Res.

[9] Kim TY, Lee JW, Kim HP, Jong HS, Kim TY (2007). DLC-1, a GTPase-activating protein for Rho, is associated with cell proliferation, morphology, and migration in human hepatocellular carcinoma.. Biochem Biophys Res Commun.

[10] Qian X, Li G, Asmussen HK, Asnaghi L, Vass WC (2007). Oncogenic inhibition by a deleted in liver cancer gene requires cooperation between tensin binding and Rho-specific GTPase-activating protein activities.. Proc Natl Acad Sci U S A.

[11] Xue W, Krasnitz A, Lucito R, Sordella R, Vanaelst L (2008). DLC1 is a chromosome 8p tumor suppressor whose loss promotes hepatocellular carcinoma.. Genes Dev.

[12] Healy KD, Hodgson L, Kim TY, Shutes A, Maddileti S (2008). DLC-1 suppresses non-small cell lung cancer growth and invasion by RhoGAP-dependent and independent mechanisms.. Mol Carcinog.

[13] Wong CM, Lee JM, Ching YP, Jin DY, Ng IO (2003). Genetic and epigenetic alterations of DLC-1 gene in hepatocellular carcinoma.. Cancer Res.

[14] Katz M, Amit I, Citri A, Shay T, Carvalho S (2007). A reciprocal tensin-3-cten switch mediates EGF-driven mammary cell migration.. Nat Cell Biol.

[15] Yam JW, Ko FC, Chan CY, Jin DY, Ng IO (2006). Interaction of deleted in liver cancer 1 with tensin2 in caveolae and implications in tumor suppression.. Cancer Res.

[16] Yuan BZ, Jefferson AM, Millecchia L, Popescu NC, Reynolds SH (2007). Morphological changes and nuclear translocation of DLC1 tumor suppressor protein precede apoptosis in human non-small cell lung carcinoma cells.. Exp Cell Res.

[17] Scholz RP, Regner J, Theil A, Erlmann P, Holeiter G (2009). DLC1 interacts with 14-3-3 proteins to inhibit RhoGAP activity and block nucleocytoplasmic shuttling.. J Cell Sci.

[18] Liao YC, Shih YP, Lo SH (2008). Mutations in the focal adhesion targeting region of deleted in liver cancer-1 attenuate their expression and function.. Cancer Res.

[19] Zhou X, Thorgeirsson SS, Popescu NC (2004). Restoration of DLC-1 gene expression induces apoptosis and inhibits both cell growth and tumorigenicity in human hepatocellular carcinoma cells.. Oncogene.

[20] Zhou X, Zimonjic DB, Park SW, Yang XY, Durkin ME (2008). DLC1 suppresses distant dissemination of human hepatocellular carcinoma cells in nude mice through reduction of RhoA GTPase activity, actin cytoskeletal disruption and down-regulation of genes involved in metastasis.. Int J Oncol.

[21] Chan LK, Ko FC, Ng IO, Yam JW (2009). Deleted in liver cancer 1 (DLC1) utilizes a novel binding site for Tensin2 PTB domain interaction and is required for tumor-suppressive function.. PLoS ONE.

[22] Kawai K, Iwamae Y, Yamaga M, Kiyota M, Ishii H (2009). Focal adhesion-localization of START-GAP1/DLC1 is essential for cell motility and morphology.. Genes Cells.

[23] Jang BC, Paik JH, Jeong HY, Oh HJ, Park JW (2004). Leptomycin B-induced apoptosis is mediated through caspase activation and down-regulation of Mcl-1 and XIAP expression, but not through the generation of ROS in U937 leukemia cells.. Biochem Pharmacol.

[24] Feng M, Huang B, Du Z, Xu X, Chen Z (2011). DLC-1 as a modulator of proliferation, apoptosis and migration in Burkitt's lymphoma cells.. Molecular biology reports.

[25] Yang XY, Guan M, Vigil D, Der CJ, Lowy DR (2009). p120Ras-GAP binds the DLC1 Rho-GAP tumor suppressor protein and inhibits its RhoA GTPase and growth-suppressing activities.. Oncogene.

[26] Erlmann P, Schmid S, Horenkamp FA, Geyer M, Pomorski TG (2009). DLC1 activation requires lipid interaction through a polybasic region preceding the RhoGAP domain.. Molecular biology of the cell.

[27] Hers I, Wherlock M, Homma Y, Yagisawa H, Tavare JM (2006). Identification of p122RhoGAP (deleted in liver cancer-1) Serine 322 as a substrate for protein kinase B and ribosomal S6 kinase in insulin-stimulated cells.. J Biol Chem.

[28] Ko FC, Chan LK, Tung EK, Lowe SW, Ng IO (2010). Akt phosphorylation of deleted in liver cancer 1 abrogates its suppression of liver cancer tumorigenesis and metastasis.. Gastroenterology.

[29] Dubash AD, Guilluy C, Srougi MC, Boulter E, Burridge K (2011). The Small GTPase RhoA Localizes to the Nucleus and Is Activated by Net1 and DNA Damage Signals.. PLoS One.

