# Supplementary figures and images for: Nuclear-Targeted Deleted in Liver Cancer 1 (DLC1) Is Less Efficient in Exerting Its Tumor Suppressive Activity Both In Vitro and In Vivo
Source: PLoS One. 2011 Sep 26;6(9):e25547. doi: 10.1371/journal.pone.0025547 (PMC3180446; doi:10.1371/journal.pone.0025547)

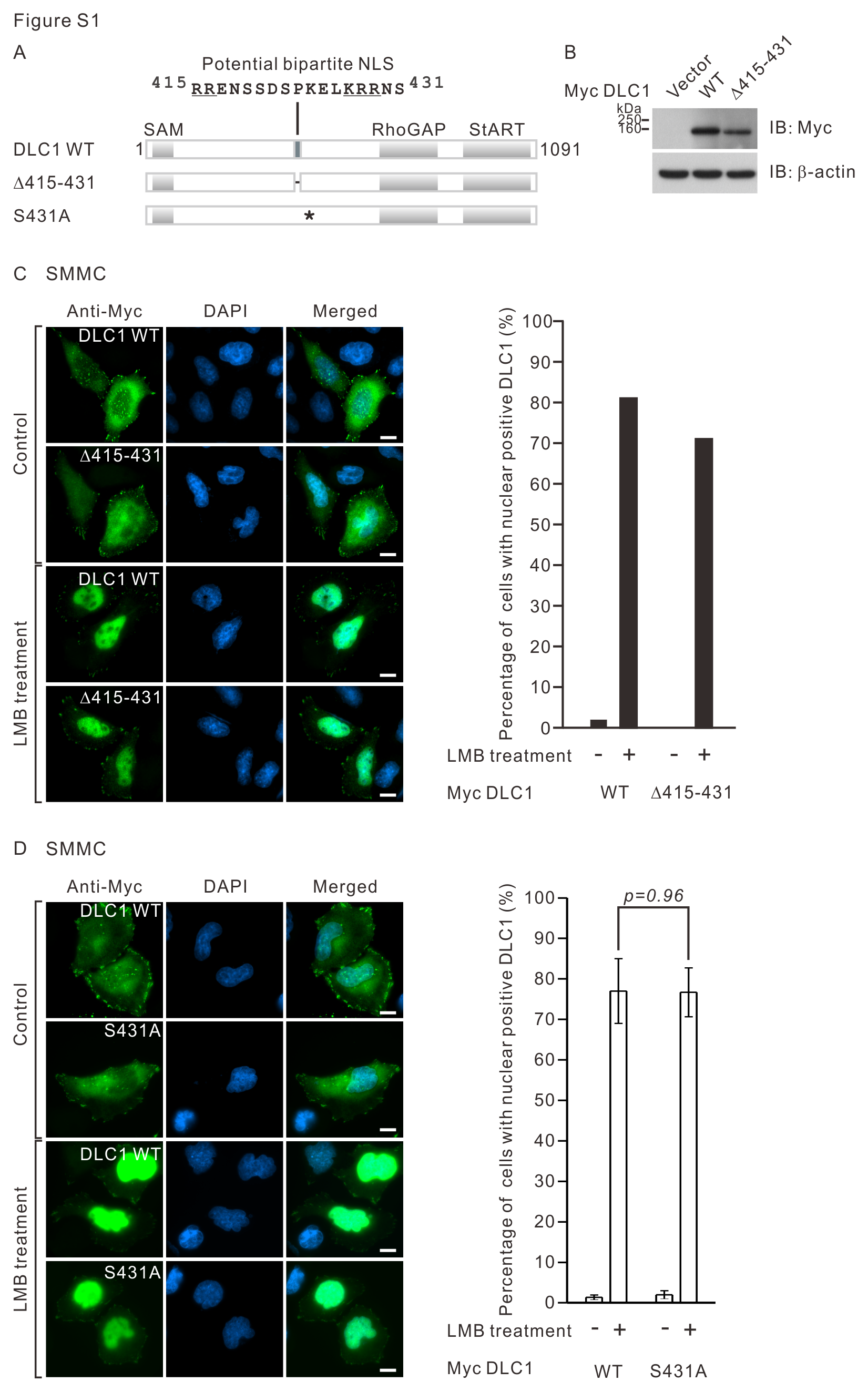

Supplement: Figure S1 — DLC1 ΔNLS (Δ415–431) could still be retained in the nucleus upon LMB treatment in SMMC–7721 cells. (A) Schematic diagram showing the proposed NLS in DLC1 as stated by Yuan et al and Schloz et al. The S431 site was proposed as a 14-3-3 docking site which modulates the DLC1 nuclear entry by Scholz et al. The structure of DLC1 ΔNLS (Δ415–431) and S431A mutants were outlined. (B) Western blotting showing the protein expression of Myc-tagged wild type DLC1 and ΔNLS mutant in transiently transfected SMMC-7721 cells. (C) Immunofluorescence staining showing the localization of wildtype DLC1 and ΔNLS in the presence or absence of LMB. DLC1 was visualized with anti-Myc antibody following by FITC conjugated antibody. Nucleus was counterstained with DAPI. The subcellular localization of DLC1 was recorded by counting at least 100 transfected cells per sample. Bar graph summarizing the subcellular localization patterns of DLC1 and the ΔNLS mutant. The results represent a duplicate of two independent experiments. (D) Immunofluorescence staining showing the localization of wildtype DLC1 and S431A mutant in cells in the presence or absence of LMB. Bar graph summarizing the subcellular localization patterns of DLC1 and the ΔNLS mutant. The results represent a triplicate of three independent experiments. Scale bar: 10 µm. (TIF) [file pone.0025547.s001.tif]

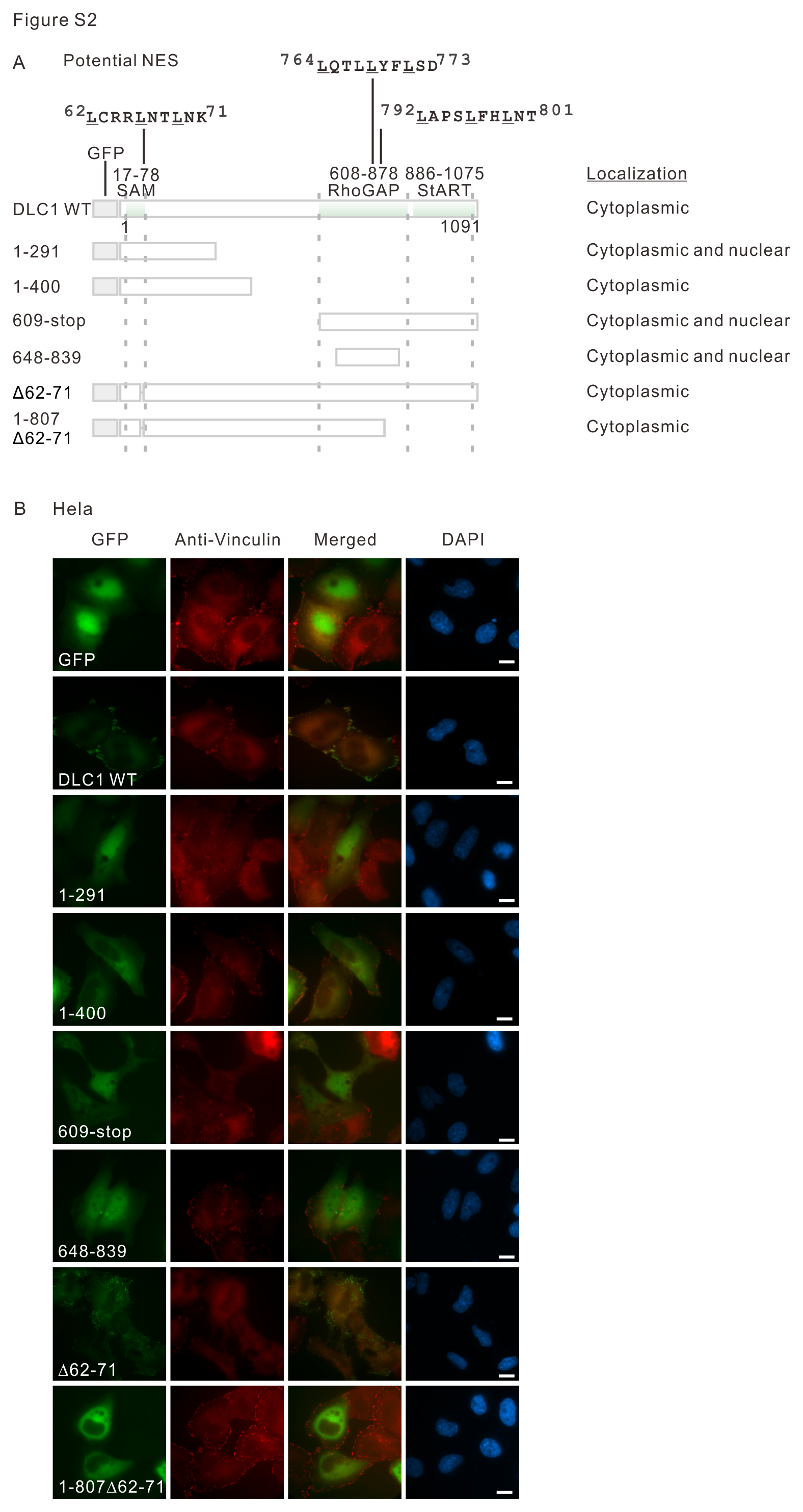

Supplement: Figure S2 — Prediction and characterization of potential Nuclear Exporting Signals (NES) in DLC1. (A) Schematic diagram showing the position of the three potential NESs in DLC1 based on the in silico search for Leucine rich motif (LXXXLXXLXX; L = Leucine; X = Any amino acids). The structure and subcellular localization of DLC1 constructs used to pinpoint the potential NES were listed. (B) (E) HeLa cells were transiently transfected with GFP-tagged DLC1 expression constructs listed in (A). Focal adhesions were counterstained with anti-vinculin antibody. Scale bar: 10 µm. (TIF) [file pone.0025547.s002.tif]

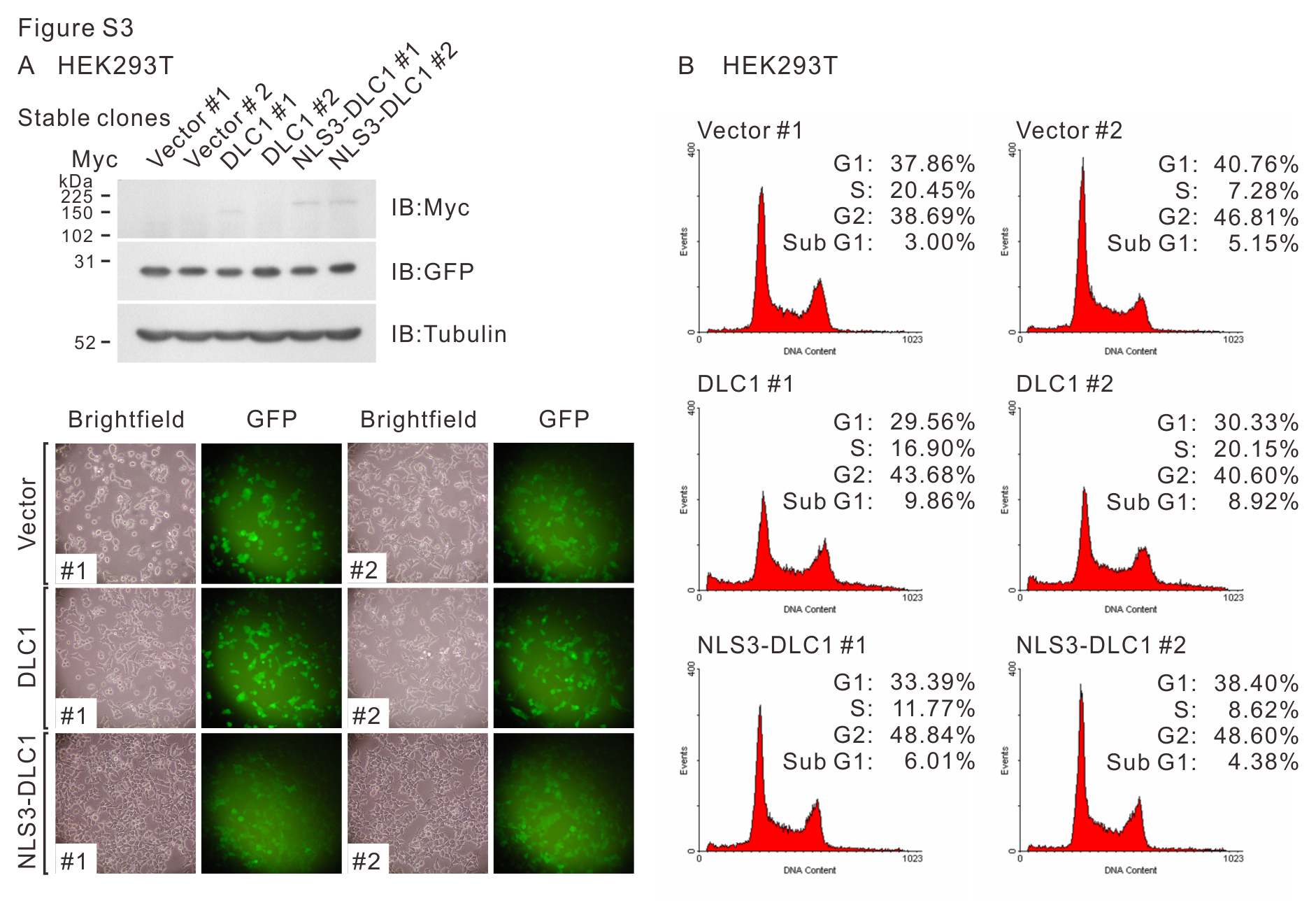

Supplement: Figure S3 — Stable expression of nuclear targeted DLC1 was less potent in inducing apoptotic subG1 cell population in HEK293T cells. (A) HEK293T cells were transduced with the indicated retroviruses. Two individual clones of each group were picked and propagated. Transduced cells were GFP positive. HEK293T cells transduced with the indicated retroviruses were lysed and subjected to immunoblotting using anti-Myc and anti-GFP antibodies. Tubulin was served as the loading control. (B) HEK293T cells transduced with the indicated retroviruses were subjected to flow cytometry analysis for propidium iodide staining. The cell cycle profiles of individual cell lines were shown. (TIF) [file pone.0025547.s003.tif]
